# A Two-Stage SEM—Artificial Neural Network Analysis of the Engagement Impact on Employees’ Well-Being

**DOI:** 10.3390/ijerph19127326

**Published:** 2022-06-15

**Authors:** Luminita Popescu, Claudiu George Bocean, Anca Antoaneta Vărzaru, Costin Daniel Avram, Anica Iancu

**Affiliations:** 1 Department of Management, Marketing and Business Administration, Faculty of Economics and Business Administration, University of Craiova, 13 AI Cuza Street, 200585 Craiova, Romania; luminita.popescu@expert.org.ro (L.P.); anica.iancu@edu.ucv.ro (A.I.); 2 Department of Economics, Accounting and International Business, Faculty of Economics and Business Administration, University of Craiova, 13 AI Cuza Street, 200585 Craiova, Romania; anca.varzaru@edu.ucv.ro (A.A.V.); costin.avram@edu.ucv.ro (C.D.A.)

**Keywords:** employee involvement, employee satisfaction, individual behavior, structural equation modeling, artificial neural network analysis

## Abstract

Employees’ engagement (EE) and well-being (WB) are considered two interesting issues by many scientific researchers and practitioners within organizations. Most research confirms a positive correlation between EE and WB. EE is an essential premise for specific dimensions of employees’ WB. At the same time, satisfied and physically and mentally healthy employees increase EE, both EE and WB thus being fundamental to individual and organizational performance. This paper aims to evaluate the relationships between EE and WB and between the dimensions of these two complex constructs. These relationships were assessed based on data obtained from a sample of 269 employees in Romania, using as a method a mix of analyses based on structural equation modeling (SEM) and artificial neural network analysis (ANN). The results highlighted a positive two-way relationship between EE and WB. Among the dimensions of EE, motivation and work environment are those that ensure a more pronounced perception of WB by the employee. Emotional WB, occupational WB, and social WB are the dimensions of WB with a significant influence on the general level of EE.

## 1. Introduction

As Professor Klaus Schwab argued at the 2012 World Economic Forum in Davos, today, the top economic ideology is shifting from capitalism to talent—a new era in which human capital has become more important for the success of countries, cities, and companies than financial capital [1]. The “engine” of this change is the generation that predominates in the current labor market, namely Generation Y (Millennials). Unlike their previous generation (Generation X), characterized by tendencies of loyalty and attachment to the organization throughout their careers, Millennials are much more willing to change their job if it does not provide a motivating work environment.

Therefore, attracting, retaining, and developing talent in organizations is one of the current challenges for both practicing managers and management science. In addition, identifying, knowing, and understanding the relationship between employee engagement (EE), satisfaction, and well-being (WB) is a significant concern in contemporary organizations. Organizations are in constant competition for new customers and markets and face the development and expansion of artificial intelligence (AI), globalization, the rapidly growing population, and, more recently, the health and economic crisis generated by the COVID-19 pandemic.

The pandemic generated by COVID-19 added to the increased psychological pressure and uncertainty in the workplace, leading to negative consequences for workers. Given the relationship between the involvement of employees and welfare, in this paper, we aim to identify the relationships established between the main factors of EE and the dimensions of employee welfare. Existing research provides insights into human resource management strategies in teamwork, providing a facilitative work environment, adopting effective leadership [2], and motivating employees to stimulate EE [3]. These measures aim to improve WB and increase the organization’s performance by involving staff. Yang et al. [4], as well as De-la-Calle-Durán and Rodríguez-Sánchez [5], showed that there is an antecedent relationship between employee EE and WB. In addition, other authors [6,7,8] have shown that EE and WB positively impact organizational efficiency, productivity, and performance.

The paper structure has six sections. After an introduction and a review of the literature, the paper proposes a methodology for researching these relationships. The Section 4 presents the results of the empirical research, and the Section 5 provides the discussions. Finally, the Section 6 concludes with research limitations and future research directions.

## 2. Literature Review

### 2.1. Employees’ EE

In general, EE is an emotional and intellectual state that causes employees to be highly attached to their work and its goals. As a result, the EE has become a management concern and a common research topic for the last three decades. Unfortunately, although many studies have been published on this topic, there is still no consensus on the significance of assessing this employee’s condition.

The study by Bailey et al. [9] highlights that the conceptualization of EE in the literature starts from the definition given in the early 1990s by Kahn [10]. Kahn [10] defined EE as the simultaneous expression of an individual’s preferred self that promotes optimal work and team connections, personal involvement (physical, cognitive, and emotional), and total active performance. Currently, EE is viewed from several perspectives involving employee involvement in various research models related to psychological, motivational, and professional issues [11,12,13]. Recent research has undergone an empirical investigation of the multidimensional framework proposed by Kahn [10], especially when EE is related to the dimensions of WB [14,15,16,17]. Shuck et al. [16] and Shuck and Reio [17] focused their research on the three distinct levels of EE proposed by Kahn: cognitive EE, emotional EE, and behavioral EE. Cognitive EE assesses employees’ significance of their work (physically, emotionally, and psychologically) and the resources needed to complete it. Emotional EE defines the amount of investment in emotional resources employees engage in the workplace. Behavioral EE is both a combination and a result of cognitive and emotional EE, which is transposed into increased effort towards organizational goals [18]. Stuck and Reio [17] conclude that EE is a series of psychological states (cognitive, emotional, and behavioral) that are transposed into the intent to act, including motivational characteristics but separate from similar (job satisfaction) or antagonistic constructs (the burnout defined by Schaufeli et al. [19]).

According to Bakker et al. [20], EE is a positive, satisfying state, which determines an affective-motivational state of professional WB; the employees involved have a high level of energy and show enthusiasm in their work. Truss et al. [21] consider EE as a passion for work, characterized by employees’ emotional, cognitive, and physical dimensions during work. The EE is based on the flexibility, change, and continuous improvement of the work environment. It allows employees to be motivated to work and care about their work. Anitha [22] found that the work environment and team relationships positively influence EE, and an engaged employee is aware of his responsibility for the organization’s goals and motivates his colleagues to achieve them.

EE means a set of positive employee behaviors that lead to superior workplace performance, consistent with the organization’s mission [23]. EE is characterized by a high level of vigor and a strong identification with a person’s work. Saks [11] concludes that EE’s dimensions are associated with individual role performance, different from organizational engagement, organizational citizenship behavior, and job involvement.

To carry out the research, starting from the results of the previous study and an approach from the perspective of human resources management, we defined EE as a construct that has four dimensions: motivation and development perceived by the company’s employees, work environment, leadership, and the employee’s loyalty to the company.

### 2.2. Employees’ WB

The notion of WB can be seen from different points of view. A macro perspective includes poverty rates, life expectancy, and environmental vectors [24]. On the other hand, WB is a concept that describes how an individual relates to his situation in life. The idea of WB has multiple dimensions: physical, emotional, and spiritual health, perception of one’s competence and purpose in life, connection and belonging to a group, optimism, and financial status. From these allegations, we notice that WB is a subjective concept: how a person perceives his situation influences the standard of living and life expectancy, level of involvement, level of performance at work, productivity, and financial success [25,26]. In addition, Vitale [27] shows that a lack of WB leads to reduced performance.

“WB is the state of comfort, health, or happiness. At work, WB is a much broader concept than personal happiness. For employees, the level of WB is related to how satisfied they are with their work and how the organization treats them, especially in their health care” [28] (p. 565). In addition, Inceoglu et al. [29] emphasize that WB supports and increases employee involvement and, therefore, organizational performance and competitiveness for the functioning of organizations.

Researchers in various fields (economists, psychologists, sociologists, and doctors) have evaluated the conditions that ensure a better life for employees and create a sense of WB [30]. Based on long-term studies and research, Rath and Harter [30] distinguish five critical areas of WB: career, social life, financial situation, health, and community. These areas interact, influence each other, and impact overall WB [26,31,32,33]. Because WB is subjective and cannot be measured by objective indicators, it is necessary to assess the perceptions of individuals invited to self-assess by completing questionnaires, conducting interviews, or observing behaviors that indicate WB dimensions [25].

The literature analysis leads us to the conclusion that there is no clear definition of employees’ WB [34,35,36,37,38,39,40,41,42]. However, the World Health Organization gives a comprehensive definition of employees’ WB—the condition of “each employee in which they understand their abilities, cope with life stress, work productively and contribute to their development in the community” [43] (p. 3). Furthermore, research indicates that psychological WB is the most crucial WB factor in the work process [44].

The model used to formulate the hypotheses of our research assumes the existence of eight dimensions of WB: emotional WB, environmental WB, intellectual WB, occupational WB, physical health, social WB, spiritual WB, to which is added the general perception of employees on WB, as defined by TINYpulse [45].

### 2.3. Relationship among Employees’ EE, WB, and Other Organizational Measures

There is still no consensus in the academic literature on the significance of EE and its operationalization. The concerns related to the investigation of the subject come mainly from the publications of practitioners and consulting firms. Still, many studies analyze the relationships between EE and other individual and organizational variables. These concerns for the study of EE are consequences at the organizational and individual levels.

Thus, a global study by Harter et al. [6] highlighted the far-reaching influence that EE and employee satisfaction have on the productivity and profitability of organizations. Although research on the relationship between EE and performance indicators has predominated in the literature, several studies have focused on the implications of EE on health and WB [46,47,48]. For example, Schaufeli et al. [19] investigated the relationship between EE and exhaustion (a negative form of WB), affecting productivity and performance. Other researchers [46] have found that emotional exhaustion and personal fulfillment are indicators of WB that depend on an individual’s psyche. In a subsequent paper, Schaufeli [49] relates several elements that determine the climate at work with factors that promote the development of EE and thus stimulate WB. Investigating the psychological environment in the workplace and associating it with EE, WB, and performance is an essential topic for much research [16,46,50,51].

An employee’s perception of the workplace climate is a variable that must be taken into account when analyzing decisions about the intensity and direction of EE [49]. The research of Shuck et al. [16] and Shuck and Reio [17] provided evidence on the link between the psychological climate at work and workers’ level of EE. On the other hand, other authors [52,53] show that raising personnel qualifications through development and motivation achieves higher EE in continual improvement in line with international standards, namely ISO 45001 and ISO 9000.

## 3. Research Design and Methodology

**Figure 1:**
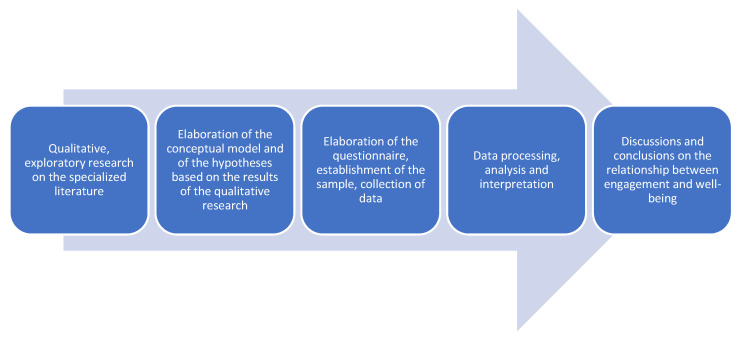
The research process. Source: Developed by the authors.

In this research, we adopted a philosophy of pragmatism, combining qualitative research with quantitative analysis. The qualitative research was exploratory and was carried out on the specialized literature, being the basis for creating the theoretical-conceptual model. In the empirical study, we used quantitative research methods, and the hypotheses were formulated based on qualitative research and were tested based on the theoretical-conceptual model. Figure 1 illustrates the research process in our paper.

**Figure 2:**
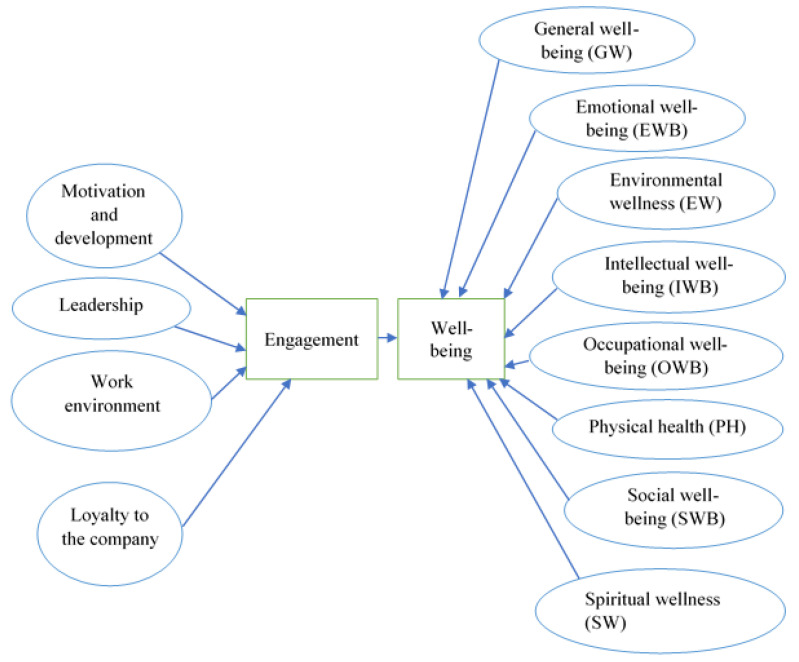
Theoretical-conceptual model regarding the relationship between EE and WB. Source: Developed by the authors.

The exploratory results undertaken in the literature led us to develop a multidimensional model of the relationship between EE and WB. The theoretical-conceptual model is presented in Figure 2.

Starting from the variables defined in the theoretical-conceptual model on the relationship between EE and WB, we developed a questionnaire consisting of three parts containing demo-socio-economic variables, variables on EE, and variables on WB. The questionnaire structure is presented in Table 1 and questionnaire items in Appendix A.

The individual variables (questionnaire items) were aggregated by calculating the leading trend indicator (arithmetic mean), obtaining aggregate variables for the four dimensions of EE (motivation and development, work environment, leadership, and loyalty to the employer), and the eight dimensions of WB (general WB, emotional WB, environmental wellness, intellectual WB, occupational WB, physical health, social WB, spiritual wellness).

The empirical study was conducted on a sample of 269 employees in Romania, using the Internet as a means of transmission, according to the methodology described by Dillman et al. [54]. The questionnaire was sent to 315 individuals, with a response rate of 85.39%. Within the sample, 40.15% are male, and 59.85% are female. Regarding the age, 7.06% are under 30 years old, 69.93% are between 31 and 55 years old, and 13.01% are over 55. In addition, 16% of respondents have secondary education and 84% have a higher degree. Over 72% of respondents have more than ten years of work experience, and over 58% have more than ten years of experience in the organization. Most respondents are subordinates, with only 7.81% being managers.

The use of self-administered questionnaires can generate a problem that may affect the relevance through the common method bias—CMB [55]. Using Harman’s single-factor test, we tested all variables by exploratory factor analysis using principal component analysis. The total variance extracted was below 50% (44.943%), proving no substantial bias effects [55].

Following the exploratory research undertaken on the specialized literature, we formulated the following hypotheses:

**Hypothesis** **1** **(H1).** *EE has a significant positive influence on WB, with a two-way relationship between the two constructs.*

**Hypothesis** **2** **(H2).** *Among the dimensions of EE, those concerning motivation and the work environment have a significant positive influence on workers’ general EE and WB.*

**Hypothesis** **3** **(H3).** *Among the dimensions of WB, environmental wellness and intellectual and occupational WB have a significant positive influence on workers’ general level of WB and workers’ general level of EE.*

To investigate the validity of the formulated hypotheses, we used quantitative research techniques: a mix of analyses based on structural equation modeling (SEM) and artificial neural network (ANN). This combination of methods used in a sequential run aims at triangulating the results obtained with SEM and strengthening them based on the NN findings. SEM is useful for determining the relationship between EE and WB, the two concepts being defined as latent variables with antecedents defined as observable exogenous variables (motivation and development, leadership, loyalty to the company being antecedents of employees’ engagement, and general WB, emotional WB, intellectual well-being, occupational WB, physical health, social WB, spiritual wellness antecedents of WB). We used ANN to identify the relationships and the influences of each variable representing a background with EE and WB. Two-stage research allows for a more precise and more accurate analysis. First, SEM indicates the intensity of the relationship between EE and WB, and ANN details the relationships between their components and general constructs.

## 4. Results

**Figure 3:**
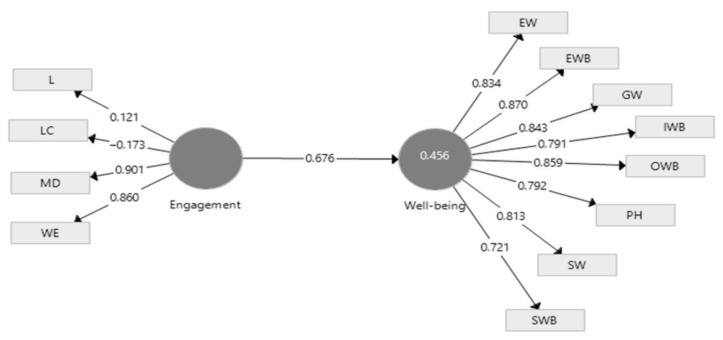
Preliminary evaluation of the theoretical-conceptual model using SEM-PLS. Source: Developed by authors using SmartPLS v3.0 (SmartPLS GmbH, Oststeinbek, Germany).

To validate the first hypothesis, we used structural equation modeling (SEM) to identify and evaluate the established relationships between the variables. The model used was created using SmartPLS v3.0 (SmartPLS GmbH, Oststeinbek, Germany), which offers capabilities to calculate partial least square (PLS). Figure 3 shows the meaning, significance, and intensity of the relationships established within the theoretical-conceptual model adopted in the research cart.

**Figure 4:**
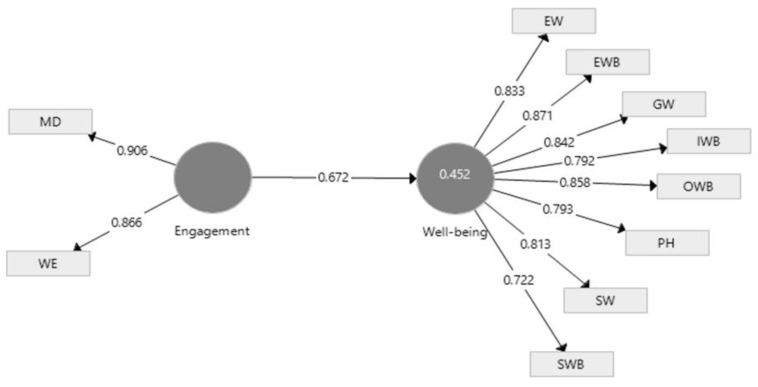
The model obtained using a selected sample. Source: Developed by authors using SmartPLS v3.0 (SmartPLS GmbH, Oststeinbek, Germany).

According to Hair et al. [56], all observable external variables must have a loading greater than 0.7 for the model to be valid. Following the analysis of the theoretical-methodological model, we noticed that two of the dimensions of EE do not fit into the model. For example, leadership and loyalty to the company make a discordant note in the EE in the case of the selected sample. Therefore, we eliminated the two dimensions by obtaining a model with a good fit. The accepted model is below (Figure 4):

The applied model presents suitable fit measures. The standardized root mean square residual (SRMR) is 0.058, while normed fit index (NFI) registers 0.937. According to Hair et al. [57], a value for standardized root mean square residual (SRMR) less than 0.08 and a value for normed fit index (NFI) above 0.9 usually are considered a good fit. The values demonstrating the model’s fitness, reliability, and validity are presented in Table 2.

Figure 4 indicates the intensity of the relationship is high, which leads us to find a significant positive influence of the EE on the WB of employees. Furthermore, the f square, which indicates the size effect, has a value of 0.823, strengthening the conclusions on the relationship between EE and WB.

The results obtained by applying the modeling of the partial least square structural equation (SEM-PLS) to the data collected among the selected sample lead us to say that the H1 hypothesis is validated. EE positively influences their WB, with a two-way relationship between the two constructs.

**Figure 5:**
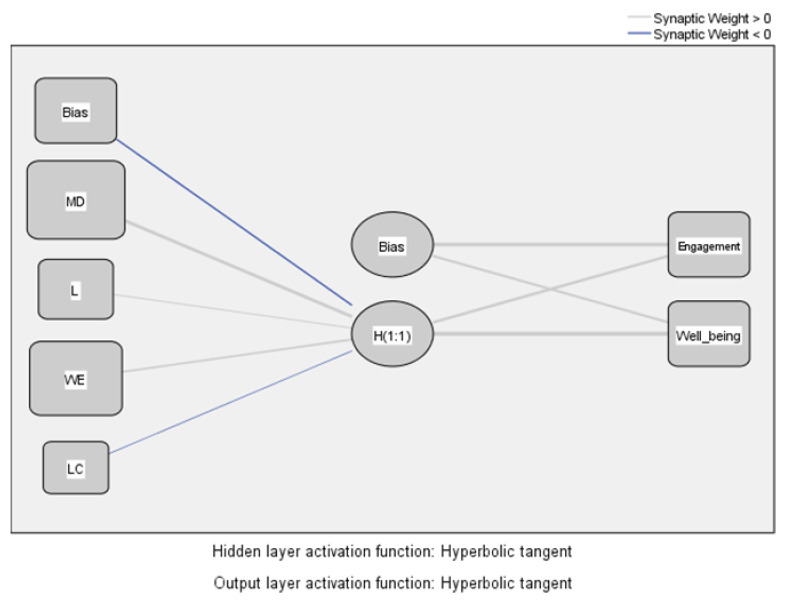
MLP model for identifying the influences of EE dimensions on the overall level of EE and WB. Source: Developed using SPSS v.20 (SPSS Inc., Chicago, IL, USA).

To verify the validity of hypotheses H2 and H3, we used artificial neural network analysis (ANN). The multilayer perceptron (MLP) model identifies the influences of EE dimensions on the overall level of employee EE and WB (Figure 5). The functions used to activate the hidden layer and the output layer are hyperbolic, the resizing method used for dependent and independent variables being data standardization.

Table 3 shows the predictions of the model values and the importance of the independent variables.

Following the analysis of Table 3 and Figure 5, we note that the dimensions of EE, motivation, and work environment significantly influence the overall level of EE and WB, confirming the results obtained in the analysis SEM-PLS.

Table 4 shows the predictions of the model values and the importance of the independent variables.

**Figure 6:**
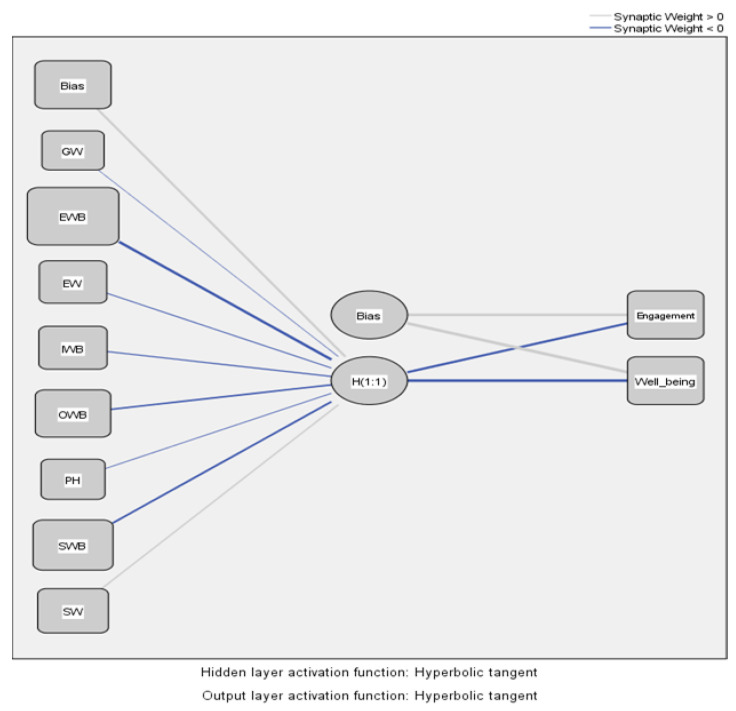
MLP model for identifying the influences of WB dimensions on the overall level of EE and WB. Source: Developed using SPSS v.20 (SPSS Inc., Chicago, IL, USA).

After analyzing Table 4 and Figure 6, we notice that the dimensions of WB, emotional WB (EWB), occupational WB (OWB), and social WB (SWB) have a significant influence on the general level of EE and WB, confirming the results obtained in the SEM-PLS analysis.

Applying the analysis of artificial neural network (ANN) on the data collected among the selected sample, it is found that hypothesis H2 is fully validated, and H3 is partially validated. Among the dimensions of EE, those related to motivation and work environment significantly influence workers’ general level of EE and WB. Among the dimensions of WB, emotional, occupational, and social WB substantially influence the general level of WB of workers and the general level of EE of workers.

## 5. Discussion

A low EE can be a negative aspect of the organization’s work due to decreased organizational performance resulting from a decrease in WB and productivity. On the other hand, an engaged employee is more than an executor of the assigned tasks. He is enthusiastic and passionate about his work, aligned with the values, and dedicated to boosting its growth, convinced that he will develop with it.

Research on EE has extended [12]. For example, many researchers have concluded that the employees involved are productive [11,18], more loyal to their current employer [6,11,16], and have more customers [58]. Other researchers have linked EE to workplace performance, mediated by workplace attitudes [15,59]. In addition, other research has linked higher levels of involvement to the growing revenue of organizations [60,61].

The results of our research provide empirical support for the hypothesis that the workplace climate influences productivity and performance. The findings support the positive emotion theory of Fredrickson [62,63] in the workplace context [64], extending the empirical work initiated by Maslach and Schaufeli [51,65] on employment as a form of heavy work investment. Our research shows that when employees perceive their work environment as positive, they experience psychological WB and a sense of personal fulfillment, which are not incompatible with an adverse psychological climate. The results obtained are similar to other researchers who support the importance of the work climate for EE and emotional and general WB [16]. Employees who psychologically appreciated the work environment reported higher levels of WB.

Lindell and Brandt [66] show that the work environment and organizational climate are psychologically important through the significant impact on employees’ overall WB and performance in the workplace. A work environment designed to support EE will promote the WB and the productivity of its employees [67]. Robertson and Cooper [68] highlight the two-way relationship between general WB and EE levels. Several studies support a positive effect of EE on WB [5,69,70]. EE is essential for self-realization, while WB results from self-fulfillment and self-improvement [71].

The organizations’ employees want to work in teams that ensure a motivational environment led by leaders who care about their WB. Employees prefer managers who support them on the professional side and on the personal side as individuals [26,27]. Research shows that employees are loyal to companies that help their employees improve or manage their WB. Within these companies, we can see more significant involvement of employees, higher labor productivity, decreased absenteeism, better customer loyalty, higher profitability, and, last but not least, lower dropout rates organization by employees [72]. EE and WB are also linked to a work environment that ensures a psychosocial climate of safety that provides for the psychological health of workers [26,73,74,75].

## 6. Conclusions

The paper assesses the level of EE and WB of Romanian employees and the relationships established between these constructs and their dimensions. The research methodology was based on a positivist-objectivist approach, and the questionnaire was the primary quantitative method. The first step of the research was a comprehensive analysis of the literature, which was the basis for developing the questionnaire and establishing research hypotheses. The evaluation of the questionnaire survey results, using descriptive statistics, structural equation modeling, and analysis of artificial neural networks allowed us to verify the validity of the hypotheses. After confirming the hypotheses, we concluded that the EE significantly influences their WB, there being a two-way relationship between the two constructs. Among the dimensions of EE, those concerning motivation and work environment substantially influence the general level of EE of workers and their WB. Among the dimensions of WB, emotional WB, occupational WB, and social WB have a significant influence on the general level of WB of workers and the general level of EE in the future activities of workers.

### 6.1. Practical Implications

The last two years, marked by the COVID-19 pandemic, have changed the dynamics of the relations between employees and employers. People’s attachment to the organization and their role in its smooth running has been subject to unprecedented realities in contemporary history. In addition to the inevitable strategic and organizational changes for the continuation of activities, the companies had to find practical solutions for coordinating, training, and controlling the activity of the staff. The introduction of health protocols and measures to increase safety at work were among the actions that led to increased employee confidence in companies. In contrast, where activity has been carried out in teleworking mode since the beginning of the crisis, the employees feel a lack of communication and interaction with the work team, leading to the alteration of the WB state.

Therefore, the pandemic crisis can catalyze essential changes at organizational and individual levels. Organizations have had to adapt their procedures, work tools, and modus operandi to the new conditions. Research has shown that EE has a positive effect on their well-being, with a two-way relationship between the two constructs. Following the research among Romanian employees, we found that the two dimensions of EE, leadership and loyalty to the company, are weak and do not participate in employees’ commitment. Therefore, they were removed from the applied model. The practical implications of the results of our application are the recommendations to the employer to take into account the well-being of employees in all its dimensions, because a satisfied employee shows a high level of EE. Employee–employer relationships involve unique benefits, different ways of involving employees, stimulation, and effective communication [76].

The COVID-19 pandemic creates a state of uncertainty among workers, as organizations have begun to ignore the psychological state of employees in a desperate attempt to reduce the losses caused by this pandemic, which compromises the involvement and WB of their employees. However, organizational resilience during such crises requires employees to be both skilled, resilient, and, most importantly, involved in the work and survival of the organization. Furthermore, employees will be more involved when they trust that the organization will protect their health.

### 6.2. Limitations and Recommendations for Future Research

Despite the online data collection protocol of Dillman et al. [54], the participation rate was not 100%. However, the response rate corresponds to the results of previous research that used questionnaires transmitted via the Internet [54]. Secondly, one of the research limitations relates to the conduct of the survey in one country, which makes the cultural aspects limit the generalizing power of the research results. Third, because data on employee perceptions of internal psychological constructs were used, it raises questions about generalization and common bias methods that may influence the reliability of research [55]. To reduce bias in the future, we propose a study that takes into account objective measures regarding the level of performance of employees. Cross-sectional models also limit establishing causal relationships from the findings. Even though SEM provides a perspective on the meaning and breadth of relationships, research based on longitudinal data is desirable in the future.

Regarding future research opportunities and beyond the need to collect longitudinal data, the model could include perspectives at the organizational level. For example, the impact of organizational resources, such as culture, climate, and human resource policies and corporate responsibility initiatives on meaning and EE, could be usefully explored to contribute to the development of management science and practice [77,78], as well as the impact of corporate responsibility initiatives.

## Figures and Tables

**Table 1:** Questionnaire structure.

Parts	Variables	Items
Part A Data about respondents	Gender (G)	One item
	Age (A)	One item
	Education (E)	One item
	Work seniority (WS)	One item
	Organizational seniority (OS)	One item
	Position (P)	One item
Part B EE	Motivation and development (MD)	Five items
	Leadership (L)	Eight items
	Work environment (WE)	Three items
	Loyalty to the company (LC)	Three items
Part C WB	General WB (GWB)	Four items
	Emotional WB (EWB)	Four items
	Environmental wellness (EW)	Three items
	Intellectual WB (IWB)	Four items
	Occupational WB (OWB)	Four items
	Physical health (PH)	Four items
	Social WB (SWB)	Four items
	Spiritual wellness (SW)	Four items

**Table 2:** Reliability and validity of the applied model.

	Cronbach’s Alpha	rho_A	Composite Reliability	AVE
EE	0.729	0.743	0.880	0.786
WB	0.928	0.931	0.941	0.667

Source: Developed by authors using SmartPLS v3.0 (SmartPLS GmbH, Oststeinbek, Germany).

**Table 3:** Predictors of the multilayer perceptron model to identify the influences of EE dimensions on the overall level of EE and WB.

Predictors		Value Predictions			The Importance of Independent Variables	
		Hidden Layer	Output Layer			
		H (1:1)	EE	WB	Importance	Normalized Importance
Input layer	(Bias)	−0.212				
	MD	0.703			0.470	100.0%
	L	0.101			0.138	29.3%
	WE	0.473			0.392	83.3%
	LC	0.000			0.000	0.1%
Hidden layer	(Bias)		0.856	0.530		
	H (1:1)		0.571	0.871		

Source: Developed using SPSS v.20 (SPSS Inc., Chicago, IL, USA).

**Table 4:** Predictors of the multilayer perceptron model for identifying the influences of WB dimensions on the overall level of EE and WB.

Predictors		Value Predictions			The Importance of Independent Variables	
		Hidden Layer	Output Layer			
		H (1:1)	EE	WB	Importance	Normalized Importance
Input layer	(Bias)	0.392				
	GW	−0.014			0.013	3.8%
	EWB	−0.551			0.335	100.0%
	EW	−0.097			0.072	21.4%
	IWB	−0.109			0.080	23.9%
	OWB	−0.195			0.157	46.7%
	PH	−0.034			0.031	9.2%
	SWB	−0.299			0.207	61.9%
	SW	0.131			0.106	31.5%
Hidden layer	(Bias)		0.870	1.157		
	H (1:1)		−0.513	−0.999		

Source: Developed using SPSS v.20 (SPSS Inc., Chicago, IL, USA).

## Data Availability

Not applicable.

## Appendix A

**Table A1:** Questionnaire items.

Part A Data about respondents	G	Male, female
	A	under 20 years, 20–30 years, 31–45 years, 46–55 years, over 55 years
	E	high school, bachelor, master, doctorate
	WS	under one year, 1–5 years, 5–10 years, 10–20 years, 20–30 years, over 30 years
	OS	under one year, 1–5 years, 5–10 years, 10–20 years, 20–30 years, over 30 years
	P	manager, subordinate
Part B EE	MD	Five items: autonomy; equitable pay; job compatibility; achievable goals; employer support
	L	Eight items: participation in decisions; managerial style; correct assessment; managerial communication; manager support; manager attention, clear objectives; open communication
	WE	Three items: teamwork; work climate; organizational communication
	LC	Three items: image, reputation, employee turnover
Part C WB	GWB	Four items: goodness; meaning and purpose; proudness; ease of work
	EWB	Four items: intense emotional and resilient; stress management; work satisfaction; work joy
	EW	Three items: comfortable environment; the impact of actions on work; safe work environment
	IWB	Four items: intellectual stimulation; decision capability; personal development; challenging work
	OWB	Four items: personal job satisfaction; manageable tasks; career satisfaction; catching work
	PH	Four items: health state; disease protection; enough rest; active presence
	SWB	Four items: healthy social life; support and respect in relationships; a sense of belonging to a group or community; job detachment
	SW	Four items: meaningful life; balance in meeting needs; fulfilment; work enjoyment
